# Surgical Assistants

**DOI:** 10.1093/jhps/hnac055

**Published:** 2023-02-16

**Authors:** Richard Field

**Affiliations:** Editor-in-Chief, the Journal of Hip Preservation Surgery

At the age of 25, my father-in-law, who recently qualified from St Mary’s Hospital Medical School [1], was posted to a small rural hospital in the Kenyan rift valley. With no more than the 10th edition of Bailey & Love’s Short Practice of Surgery [2], illuminated by a kerosene lamp [3], he learned to operate under the eagle-eyed guidance of his surgical assistant, the aged hospital orderly. When I started my surgical career, my father-in-law advised me never to underestimate the importance of the surgical assistant. How right he proved to be. There is only so much that two hands can achieve, and few things have given me as much pleasure as the four-handed dance that my assistant and I have learned to perform. In Australia, retired surgeons serve as surgical assistants to their successors, and I marvel at the brilliance of this system. In my travels, I have noticed that many of the finest surgeons work with an assistant who has found his or her way into the operating theatre by the most unlikely route. Just as some surgeons are blessed with ‘magic hands’, it is only when the surgeon finds a similarly talented assistant that they achieve their finest work.

Sadly, the remuneration that surgical assistants enjoy, in the United Kingdom [4], often fails to recognise or reflect the value of their role in our operating rooms. The best surgical assistants are intuitively able to make surgery easier, faster and more elegant but, few have any experience or interest in academic research [5, 6] and, those who do, are unlikely to be funded to undertake the additional work needed to gather, process and report clinical data. Many of our most innovative and academic colleagues attract trainees who displace the surgical assistants and oblige their trainer to work with third and fourth hands that are focused on becoming the surgical lead. At the Journal of Hip Preservation Surgery (JHPS), we are aware that there is a paucity of literature on the role played by surgical assistants, their influence on the use of operating room time and the outcome of our interventions. We invite you to share studies related to the involvement of surgical assistants and other operating room personnel in hip preservation surgery.

Over the next decade, augmented reality systems will be adopted to enhance surgical training [7, 8], robotic-assisted surgery will become commonplace in our operating rooms [9, 10], the role of our surgical assistants will evolve and a new generation of computer-savvy technicians will join our teams. The value of these new technologies will need to be assessed and justified both for their economic [11, 12] and clinical benefits. JHPS would welcome manuscripts focusing on these changes, and we look forward to disseminating evidence to guide the hip preservation community as new technologies become available.

In JHPS Issue 9.4, the costs incurred in providing periacetabular osteotomy surgery are explored by Joel Williams and his colleagues [13] at the Rush University Medical Center in Chicago. The paper is interesting both for the magnitude of the costs incurred in providing this surgery in the United States and because the authors have analysed the different elements of the cost of this intervention. The authors demonstrate that under the payment and reimbursement system used in the United States, provision of periacetabular osteotomy is a clinically effective intervention that should be recognised as a robust source of income for health-care providers who are increasingly cost and profit-conscious.

Issue 9.4 also includes a study provided from members of the Physiatry team and a Radiologist at the Hospital for Special Surgery [14], in New York. Physiatry is a specialty that includes Physical Medicine and Rehabilitation physicians. The authors have demonstrated that magnetic resonance imaging scans can be used to measure proximal femoral geometry with comparable accuracy to computed tomography without exposing patients to X-rays. This work comes from physicians who investigate and provide non-surgical treatment for patients who also seek our help and serves as a useful reminder that Physiatrists, Sports Physicians and allied clinical specialists are generating research that is of equal value to hip preservation surgeons.

Ben Domb’s team at the American Hip Institute Research Foundation in Chicago has addressed the challenge of patients who present with apparent lateral joint space narrowing that occurs with ossification of the labral tissue [15]. Their work demonstrates that this subgroup of patients can benefit from arthroscopic recession of the acetabular rim and labral reconstruction. The introduction of labral reconstruction remains a relatively new intervention, and the authors provide welcome evidence that hip preservation can provide a viable alternative to hip replacement for these patients.

I hope that you enjoy all the papers in JHPS 9.4 and that you will be inspired to share your work with us in future JHPS issues.
